# The Impact of Glucagon-Like Peptide-1 on Bone Metabolism and Its Possible Mechanisms

**DOI:** 10.3389/fendo.2017.00098

**Published:** 2017-05-03

**Authors:** Chenhe Zhao, Jing Liang, Yinqiu Yang, Mingxiang Yu, Xinhua Qu

**Affiliations:** ^1^Department of Endocrinology, Zhongshan Hospital, Fudan University, Shanghai, China; ^2^Department of Orthopedics, Shanghai Key Laboratory of Orthopaedic Implants, Shanghai Ninth People’s Hospital, Shanghai Jiaotong University School of Medicine, Shanghai, China

**Keywords:** glucagon-like peptide-1, osteogenesis, bone resorption, osteoporosis, diabetes mellitus

## Abstract

The impact of antidiabetic drugs on bone metabolism is drawing increasing attention due to the discovery of a correlation between type 2 diabetes mellitus (T2DM) and osteoporosis. Glucagon-like peptide-1 (GLP-1) receptor agonists are a novel and promising class of drugs for T2DM, which may also have clinical applications in bone tissue disorders. This review examines the impact of GLP-1 on bone metabolism, including enhancement of bone mineral density and improvement of bone quality. However, the precise effect of GLP-1 on fracture risk has not been unambiguously defined. This review also summarizes our current understanding of the mechanisms by which GLP-1 affects bone metabolism. GLP-1 may act on bone by promoting bone formation, inhibiting bone resorption, and affecting the coordination of the two processes. We describe molecular pathways and proteins, such as Wnt and calcitonin, that are associated with GLP-1 and bone tissue. The specific processes and related molecular mechanisms of the effects of GLP-1 on bone metabolism need to be further explored and clarified.

## Introduction

Diabetes mellitus often leads to the development of osteoporosis, a degenerative bone disorder. Both type 1 diabetes mellitus (T1DM) and type 2 diabetes mellitus (T2DM) can affect bone mineral density (BMD) and the risk of bone fractures (1). GLP-1 receptor agonists (GLP-1RAs) are a novel class of drugs for T2DM (2); they stimulate insulin secretion, increase β cell mass, and suppress glucagon secretion (3–5). Currently, four such drugs available in the United States: exendin-4 or its synthetic version exenatide, liraglutide, albiglutide, and dulaglutide. Various products are in different stages of research and development (2, 6–8). The effectiveness of these drugs has led to increasing interest in the mechanisms underlying their effects on bone metabolism. It has been reported that glucagon-like peptide-1 (GLP-1) can enhance BMD and improve bone quality, and the relationship between GLP-1 and bone fractures is still under investigation. GLP-1 can promote bone formation and inhibit bone resorption, but the specific process and related molecular pathways are still not completely understood (9–11). This review summarizes the current state of research into the impact of GLP-1 on bone metabolism and its possible mechanisms.

## The Impact of GLP-1 on Bone Metabolism

### The Impact of GLP-1 on BMD

Glucagon-like peptide-1 may enhance BMD. In an animal experiment, GLP-1 and exendin-4 (a GLP-1 mimetic) reversed the decrease in bone mass of femurs and vertebrae in hyperlipidic and hypercaloric Wistar rats (9). In a clinical trial, 61 T2DM patients were divided into three groups randomly: liraglutide 1.8 mg/day or liraglutide 1.2 mg/day or glimepiride 8 mg/day. This research showed no significant differences in BMD among groups in drug use at 52 or 104 weeks (12). The 24-week use of exenatide in newly diagnosed and treatment-naive patients with T2DM presented no influence on BMD (13). However, 69 T2DM patients receiving metformin were also given either exenatide (*n* = 36) or insulin glargine (*n* = 33). Only patients in the former group experienced significant lowering of body weight and had maintained BMD levels, after 44 weeks of treatment (14). As weight loss results in bone loss (15), and insulin administration may contribute to reduction in bone resorption (16). This result implied that exenatide, in comparison with insulin, may promote BMD while facilitating weight reduction.

### The Impact of GLP-1 on Bone Quality

Glucagon-like peptide-1 may improve bone quality. In ovariectomy (OVX)-induced osteoporosis in aged rats, exendin-4 enhanced bone strength and prevented exacerbation in trabecular microarchitecture (10). In another study, the percent bone volume (BV/TV) and trabecular number (Tb.N) were lowered in diabetic and insulin-resistant (IR) rats, while their trabecular separation (Tb.Sp), trabecular bone pattern factor (Tb.Pf), and structure model index increased, indicating an elevated level of anisotropy in trabecular bone and destruction of the normal bone structure. It was found that GLP-1 could reduce these trends and restore normal bone structure (17). In streptozotocin-induced T1DM Swiss TO mice, liraglutide improved mechanical properties in bone tissue, but failed to reverse cortical microstructure degradation or improve whole bone mechanical properties (18). Furthermore, GLP-1 and glucose-dependent insulinotropic polypeptide double incretin receptor knockout (DIRKO) mice had increased trabecular bone mass and a higher trabecular number compared to wild-type mice, with reduced bone outer diameter, cortical thickness, and cortical area. Mechanical properties of bone matrix were also affected in these mice as evidenced by decreases in yield stress, ultimate stress, and post-yield work-to-fracture in DIRKO mice at the level of whole bone. At the tissue level, the maturity level of collagen was 9% lower in DIRKO mice, which contributed to the lowering of maximum load, hardness, and dissipated energy (19).

### The Impact of GLP-1 on Bone Fractures

The relationship between GLP-1 and the risk of bone fractures has not been fully elucidated. A population-based cohort study using data from the Clinical Practice Research Datalink database (2007–2012) concluded that GLP-1RAs were not related to a decreased risk of bone fractures, independent of dose accumulation and the type of GLP-1 drug (exenatide or liraglutide) used (20). And a case–control study also presented a same conclusion (21). Similarly, a meta-analysis has studied randomized clinical trials (RCTs) comparing GLP-1RAs and other antidiabetic drugs versus a placebo in T2DM patients (>24 weeks). It revealed that GLP-1RAs failed to reduce the risk of bone fractures compared to other antidiabetic agents [OR = 0.75, 95% confidence interval (CI) 0.28–2.02, *P* = 0.569]. However, with the lack of data on bone condition, including BMD, microarchitecture, bone quality, calcium, and phosphorus levels at baseline in this study, the results should not be taken as definitive. Furthermore, GLP-1RA is related to decreased body weight, which is unfavorable for bone fractures as weight loss induces mechanical loading and bone mass decrease. These disadvantages can obscure the potential protective effects of GLP-1RA. Additionally, GLP-1RA is associated with gastrointestinal adverse events, which may cause absorption of minerals and nutrients and thereby interfere with the beneficial function of GLP-1RA on bone physiology (22).

It is possible that different GLP-1 agents have divergent effects on bone fractures, as shown in another meta-analysis of RCTs that ended in December 2013. It indicated that liraglutide significantly reduced the risk of bone fractures (OR = 0.38, 95% CI 0.17–0.87), whereas exenatide increased the risk of bone fractures (OR = 2.09, 95% CI 1.03–4.21), compared to placebo or other antidiabetic drugs. Clues exist that may help explain the distinct results of liraglutide and exenatide. First, differences in molecular structure result in dissimilar pharmacokinetic profiles. Because liraglutide shares 97% homology with GLP-1 while exenatide only shares 50% of GLP-1, it is inferred that liraglutide is more likely to mimic the function of endogenous GLP-1. Second, exenatide tends to cause more weight loss and lower glucose control than liraglutide, which may result in a higher risk of bone fractures (11). In summary, current research is inadequate to explain the effects of GLP-1 and related drugs on bone fracture risk. Furthermore, the incidence of bone fracture is only recorded as adverse reactions instead of being part of routine data collection, which may result in less than complete information. The published animal and human studies on the impact of GLP-1 on bone metabolism are described in Table 1.

**Table 1:** **Published animal and human researches on the impact of GLP-1 on bone metabolism**.

Reference	Subjects and design	Main results (BMD, bone quality, and fracture risk)
**Animals**		

Yamada et al. (23)	GLP-1r knockout mice vs. WT mice	BMD: GLP-1r^−/−^ mice presented lowered cortical BMD
		Bone quality: GLP-1r^−/−^ mice presented diminished bone flexural rigidity

Nuche-Berenguer et al. (17)	T2D rats, IR rats, and normal rats	BMD: GLP-1 had a tendency for increasing BMD in T2D and IR rats though the differences did not reach statistical significance
	GLP-1 vs. saline (3 days by osmotic pump)	Bone quality: GLP-1 alleviated enhanced anisotropy and normalized damaged trabecular bone structure in T2D and IR rats (resulted in a reduction in Tb.Sp, Tb.Pf, and SMI)

Nuche-Berenguer et al. (24)	T2D rats, IR rats, and normal rats	BMD: exendin-4 had a tendency for increasing BMD in T2D and IR rats though the differences did not reach statistical significance
	Saline vs. exendin-4 (0.1 nmol/kg/h through osmotic pump for 3 days)	Bone quality: exendin-4 might normalize the damaged trabecular structure in IR and T2D rats (resulted in a reduction in Tb.Sp, Tb.Pf, and SMI)

Nuche-Berenguer et al. (9)	Wistar HL rats with GLP-1 vs. with exendin-4 vs. with saline vs. normal rats	BMD: GLP-1 and exendin-4 improved lowered BMC and BMD of the femur and lumbar spine of HL rats

Kim et al. (25)	4-week-old male T2D OLETF rats with saline vs. OLETF rats with exendin-4 (5 nmol/kg twice a day for 3 weeks) vs. LETO control rats with saline	BMD: exendin-4 increased BMD of the femurs in OLETF rats compared to the other two groups

Ma et al. (10)	12-month-old female Sprague-Dawley rats	BMD: exendin-4 increased BMC (10 µg/kg/day) and even increased BMD dose dependently of the femur and lumbar spine
	Sham-operated group vs. OVX with vehicle vs. OVX with 17β-estradiol (25 μg/kg/day) vs. OVX with exendin-4 (1, 3, or 10 µg/kg/day)	Bone quality: exendin-4 (3 μg/kg/day) improved bone trabecular microarchitecture similarly, as compared to estradiol. It also improved bone strength of OVX rats

Mabilleau et al. (26)	Male GLP-1r knockout mice vs. control wild-type mice	Bone quality: GLP-1r knockout mice presented damaged bone strength and quality

Sun et al. (27)	2-week-old male diabetic GK rats and age-matched male Wistar rats	BMD: liraglutide reversed the lowered cortical and trabecular BMD in GK rats
	GK rats with daily subcutaneous liraglutide injection (0.4 mg/kg/day) vs. GK rats with saline vs. Wistar control rats	Bone quality: liraglutide ameliorated abnormal cortical and trabecular bone microarchitecture in GK rats

Mansur et al. (18)	STZ-induced diabetic male Swiss TO mice	Bone quality: liraglutide improved mechanical properties in bone tissue, but there were no significant results in reversing cortical microstructure degradation or improving whole bone mechanical properties
	GIP (25 nmol/kg) vs. liraglutide (25 nmol/kg) vs. saline	

Sun et al. (28)	5-month-old female non-diabetic and OVX Wistar rats	BMD: exendin-4 increased BMD
		Bone quality: exendin-4 improved trabecular structure and reduced trabecular spacing both in the femur and lumbar vertebrae
	Sham + vehicle vs. OVX + vehicle vs. OVX + exendin-4 (20 μg/kg/day)	Fracture risk: exendin-4 might have little influence on the mechanical resistance to fracture in the femur

Lu et al. (29)	5-month-old female Wistar rats	BMD: liraglutide increased BMD both in the femur and lumbar vertebrae
	Sham + saline vs. OVX + saline vs. OVX + liraglutide (0.6 mg/day)	Bone quality: liraglutide improved trabecular structure and reduced trabecular spacing both in the femurs and lumbar vertebrae

Pereira et al. (30)	12-week-old ovariectomized female C57BL/6NCrl mice	BMD: both drugs improved trabecular bone mass
	Liraglutide vs. exenatide vs. saline for 4 weeks	Bone quality: both drugs improved bone structure and connectivity, but had no effect on cortical architecture. Both increased osteoclast surfaces but only exenatide enhanced osteoclast number *in vivo*

Mieczkowska et al. (19)	26-week-old DIRKO mice and wild-type control mice	BMD: DIRKO mice showed higher trabecular bone mass and lower cortical bone mass
		Bone quality: DIRKO mice presented increased trabecular number but reduced cortical strength. They also showed decreased collagen maturity at the tissue level and resulted in exacerbation in bone mechanical response

**Human**		

Bunck et al. (14)	69 metformin-treated T2DM patients	BMD: exenatide was not related to significant changes of total BMD and serum markers of bone metabolism, despite a significant body weight decrease. And there was no difference between two groups in the endpoint BMD
	Exenatide vs. titrated insulin glargine for 44 weeks	

Mabilleau et al. (22)	A meta-analysis, 28 RCTs were identified	Fracture risk: the administration of GLP-1RA was not associated with reduced fracture risk compared to the use of other antidiabetic drugs
	Either a GLP-1RA use vs. another antidiabetic drug use in T2DM patients for at least 24 weeks	

Su et al. (11)	A meta-analysis, 16 RCTs were identified	Fracture risk: liraglutide might reduce the risk of bone fractures while exenatide might increase the risk of bone fractures
	Liraglutide or exenatide use vs. placebo or other diabetic drugs	

Driessen et al. (20)	A population-based cohort, T2DM patients with at least on prescription for NIAD	Fracture risk: GLP-1 RA administration was not related to decreased risk of fractures compared to other antidiabetic drugs users
	GLP-1RA users vs. never GLP-1RA users	

Iepsen et al. (31)	RCT, 37 healthy obese women aged 46 ± 2 years	BMD: the use of liraglutide reduced the loss of total and arm-leg BMC compared to control group
	With or without liraglutide (1.2 mg/day) for 52 weeks (after a low-calorie-diet-induced 12% weight loss)	

Driessen et al. (21)	A case–control study	Fracture risk: GLP-1 RA use (current, recent, or past) was not related to reduced fracture risk as compared to NIAD users
	NIAD users vs. GLP-1 RA users	

Gilbert et al. (12)	61 T2DM patients aged 19–79 years	BMD: there were no apparent differences between groups in mean total BMD
	24-week use of exenatide vs. insulin vs. pioglitazone	

Li et al. (13)	62 newly diagnosed and treatment-naive patients with T2DM	BMD: exenatide had no influence on BMD
	24-week use of exenatide vs. insulin vs. pioglitazone	

*GLP-1r, glucagon-like peptide-1 receptor; WT, wild-type; T2D or T2DM, type 2 diabetes mellitus; IR, insulin-resistant; Tb.Sp, trabecular separation; Tb.Pf, trabecular bone pattern factor; SMI, structure model index; HL, hyperlipidemic; BMC, bone mineral content; OLETF rats, Otsuka Long–Evans Tokushima Fatty rats; LETO rats, Long–Evans Tokushima Otsuka (LETO) rat; STZ, streptozotocin; OVX, ovariectomized; GK rats, Goto-Kakizaki rats; DIRKO, double incretin receptor knockout; GLP-1RA, glucagon-like peptide-1 receptor agonist; NIAD, non-insulin antidiabetic drug; GLP-1, glucagon-like peptide-1; BMD, bone mineral density*.

## The Potential Mechanisms of the GLP-1 Effects on Bone Metabolism

**Figure 1:**
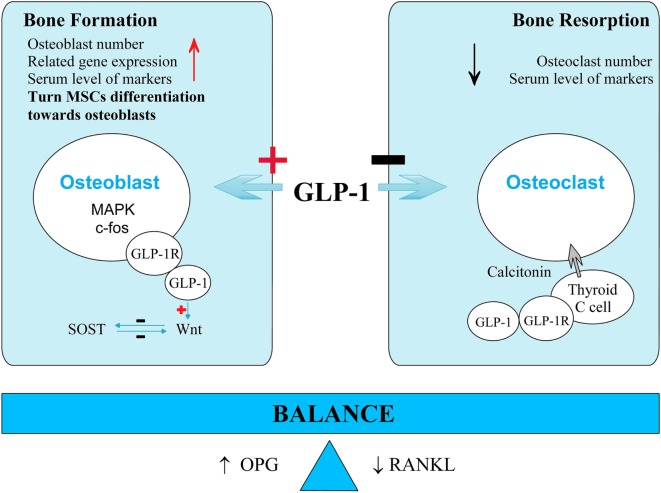
**Potential mechanism of glucagon-like peptide-1 (GLP-1) on bone metabolism**. GLP-1 promotes bone formation and inhibits bone resorption. For bone formation, GLP-1 results in increasing osteoblast number, gene expression related to bone formation and serum level of bone formation markers. GLP-1 might bind to its receptor on osteoblast and its function is possibly mediated by mitogen-activated protein kinase (MAPK) pathways, Wnt pathways, or c-fos transcription promotion. GLP-1 also turns mesenchymal stem cell (MSC) differentiation from adipocytes toward osteoblasts. For bone resorption, GLP-1 also results in decreasing osteoclast number and serum level of bone resorption markers. But GLP-1 might act through a calcitonin-dependent way in thyroid C cells. Furthermore, GLP-1 increases OPG expression while decreases receptor activator for nuclear factor-κB ligand (RANKL) expression. GLP-1 helps to maintain the balance between bone formation and bone resorption.

The continuous cycle of bone formation and bone resorption in osseous tissue maintains normal bone quality and bone mass. GLP-1 affects both parts of this cycle. Potential mechanisms of GLP-1 are described below and in Figure 1.

### Promotion of Bone Formation and Possible Mechanisms

#### GLP-1 Promotes Bone Formation

Glucagon-like peptide-1 increases the number of osteoblasts. Osteoblast number on the surface of trabecular bone was observed by a histological analysis that demonstrated a significant increase after 16 weeks of exendin-4 use in OVX rats (10). This result was also confirmed in another study (32).

Glucagon-like peptide-1 promotes the expression of genes related to bone formation. Runx2 encodes osteoblast-specific transcription factor 2, which is a transcriptional activator of osteoblast differentiation (33). Furthermore, alkaline phosphatase (ALP), collagen type 1 (Col 1), and osteocalcin (OC) are common bone formation markers (34); all are upregulated. In old rats with OVX-induced osteoporosis, 16 weeks of exendin-4 administration led to a rise in Runx2, ALP, Col 1, and OC mRNA levels (10). In another report, the use of GLP-1 significantly increased the mRNA level of OC in normal, T2DM, or IR rats (17). The same result was shown in the femurs of Goto-Kakizaki rats in liraglutide use (27). GLP-1 also exerted a similar effect on osteoblastic MC3T3-E1 cells (35). These data further demonstrate, at the gene expression level, that GLP-1 promotes bone formation in various ways.

Glucagon-like peptide-1 is associated with increased serum levels of bone formation markers. The use of exendin-4 in old OVX rats increased the serum levels of several bone formation markers, including ALP, OC, and N-terminal propeptide of type I procollagen (P1NP) (10). After 52 weeks of treatment with liraglutide, healthy obese women experienced serum P1NP level increases of 16% on average (31).

#### GLP-1 Promotes Bone Formation by Lowering Glucose Level

It was indicated that hyperglycemia was negatively associated with lumbar BMD (36). It has been demonstrated that endogenous GLP-1 could help maintain a normal glycemic level acting through GLP-1 receptors (37). GLP-1 controls the glucose level by stimulating insulin secretion, inhibiting glucagon secretion, and modulating gastric emptying, thus contributing to an enhancement of bone formation (3–5).

#### The Expression of GLP-1 Receptor on the Osteoblast Function

It has been established that GLP-1 is involved in important physiological processes through binding to the GLP-1 receptor, which is a cAMP-linked G protein-coupled receptor found in the pancreas and many extra pancreatic tissues. In a study focused on the widely studied mouse osteoblastic MC3T3-E1 cell, the expression of GLP-1 receptor was discovered to be regulated in accordance with glycemic level (38). In another study, it was established that GLP-1 receptors varied in their expression in different stages of osteoblastic cell lines. It can be deduced that the expression level of GLP-1 receptor on osteoblasts decreases with the process of osteoblast maturation (39). Furthermore, an enhanced expression of the GLP-1 receptor gene was observed during the osteogenic differentiation process of adipose-derived stem cells, which have the potential for multiple types of differentiation, including osteogenic differentiation. This result demonstrates that GLP-1 may play a role in the osteogenic differentiation of bone tissue (40).

It is notable that in a study performed by Bernardo, some human GLP-1 could bind to a functional receptor in rat MC3T3-E1 cells with a dependence on time and temperature, but independent of the type of cAMP-linked G protein receptor. To be more specific, the GLP-1 receptor with an attached GLP-1 molecule had the function of instantly hydrolyzing glycosylphosphatidylinositols (GPIs), which generated short-lived inositolphosphoglycans (IPGs), and promoted phosphatidylinositol-3 kinase (PI3K) and mitogen-activated protein kinase (MAPK) activities (35). Another study also demonstrated that GLP-1 participated in the same process in liver and muscle (41). Exendin-4 could also induce the hydrolysis of GPIs but was delayed compared to the natural human GLP-1 amide (35). These results show that GLP-1 might act directly through the GPI/IPG-coupled receptor on osteoblastic MC3T3-E1 cells.

#### The Pathways of GLP-1 in Promoting Bone Formation

Wnt pathways may play a significant role in how GLP-1 promotes bone formation. It has been reported that the Wnt pathway was impaired in diabetic rats and that exendin-4 treatment enhanced bone formation (24). The canonical Wnt pathway, which includes low density lipoprotein receptor-related protein 5/6, β-catenin, GSK-3β, and T cell factor (42), promotes osteoblast differentiation and maturation (43); GLP-1 is a direct activator of this pathway (44). Sclerostin, encoded by the SOST gene, is secreted by osteocytes and suppresses bone formation. It binds to bone morphogenetic proteins and negatively affects bone formation by inhibiting ALP activity, type I collagen synthesis, and mineralization (45). Sclerostin also inhibits the Wnt/β-catenin signaling pathway (46). Furthermore, it has been observed that exendin-4 reduced the mRNA and protein levels of SOST/sclerostin in osteocyte-like MLO-Y4 cells and also decreased the serum sclerostin level in T2DM Otsuka Long–Evans Tokushima Fatty rats. The study further proposed that exendin-4 might bind to GLP-1 receptor, mediated by protein kinase A (PKA), and act on the Wnt/β-catenin pathway in osteocytes in order to reduce sclerostin expression and promote bone formation (25).

There are other possible pathways by which GLP-1 could affect bone formation. It has been proposed that GLP-1 could promote c-Fos transcription in osteoblasts in combination with ATP, facilitating the participation of GLP-1 in bone turnover (47). It has been shown that exendin-4 enhanced the proliferation and differentiation of osteoblasts partly mediated by MAPK pathways, including ERK1/2, p38, and JNK pathways (48). It was also proposed that liraglutide regulated MC3T3-E1 cells differentiation mediated by adenosine monophosphate-activated protein kinase/mammalian target of rapamycin signaling (49).

### Suppression of Bone Resorption and Possible Mechanisms

#### GLP-1 Inhibits Bone Resorption

Glucagon-like peptide-1 has an impact on the number and functioning of osteoclasts. Osteoclast number on the surface of trabecular bone was observed by histological analysis, which demonstrated a significant decrease after 16 weeks of exendin-4 use in OVX rats (10). When ovariectomized mice were treated with liraglutide, exendin-4, or saline, it was observed that the osteoclast number was increased only in the mice that received exendin-4 and a high dose of liraglutide. Although the result was contrary to a previous study, which might partly result from different animal species, assessment method for osteoclast number and drug dose, the bone resorption efficiency of osteoclasts declined in this experiment, leading to decreased bone resorption overall (30).

Glucagon-like peptide-1 administration is associated with reduced serum levels of bone resorption markers. In old rats with OVX-induced osteoporosis, exendin-4 decreased the concentration of C-terminal cross-linked telopeptides of type I collagen (CTX-1) and the urinary deoxypyridinoline (DPD)/creatinine ratio (10). However, 52 weeks of treatment of healthy obese women with liraglutide did not significantly affect serum CTX-1 levels (31). Speculation continues on the inhibitory effect of GLP-1 on bone resorption.

#### GLP-1 Decreases Bone Resorption through a Calcitonin-Dependent Pathway

Glucagon-like peptide-1 receptor is expressed in thyroid C cells and can promote the secretion of calcitonin through a cAMP-mediated pathway in these cells (50, 51). Exendin-4 treatment caused a higher mRNA level of thyroid calcitonin in wide-type mice, whereas GLP-1r^−/−^ mice had decreased mRNA levels of calcitonin. Furthermore, GLP-1r^−/−^ mice had an enhanced level of urinary DPD, which indicated increased bone resorption. When these GLP-1r^−/−^ mice were treated with calcitonin, the increase in urinary DPD concentration was alleviated. Therefore, it can be concluded that GLP-1 inhibits bone resorption in a calcitonin-dependent way (23).

However, the results from preclinical studies have not necessarily extended to success in clinical studies. In rodents, the GLP-1 receptor expression level on thyroid C cells is high, whereas in humans, the expression of the receptor is lower. The sensitivity of the response of human TT thyroid C cells to GLP-1 that resulted in producing cAMP and releasing calcitonin was different from the rat C cell lines MTC 6–23 and CA-77. Human TT thyroid C cells were unexpectedly less responsive to GLP-1 (52). In general, GLP-1 may inhibit bone resorption *via* a calcitonin-dependent pathway, but this hypothesis still needs to be explored further.

### The Impact of GLP-1 on the Balance between Bone Formation and Bone Resorption

Normal bone metabolism in humans involves both bone formation and bone resorption in a balanced state of equilibrium. These dynamic processes involve the bone multicellular unit composed of osteoblasts, osteoclasts, and osteocytes within bone matrix (53). Osteoblasts and adipocytes are derived from mesenchymal stem cells (MSCs) (54). Liraglutide has been found to influence MSC differentiation toward osteoblasts rather than adipocytes (29). In a further exploration of the molecular mechanisms of this effect, it was revealed that GLP-1 increased hMSC proliferation, inhibited the process of their early adipogenesis, and reduced cell death in them. In this study, it was also pointed out that two potential signaling pathways involved in hMSC differentiation into adipocytes might be the targets for GLP-1: MAPK and PKC pathways (55). It has also been speculated that GLP-1 directed the differentiation tendency *via* MAPK and Wnt signaling pathways to promote Runx2 activity (28). In another research, it was demonstrated that GLP-1 promoted MSC differentiation direction into osteoblasts though acting on PKA/β-catenin and PKA/PI3K/AKT/GSK3β pathways (32). It was also proposed that the target might be extracellular signal-regulated kinase signaling pathway (56). Furthermore, two studies revealed new molecular mechanisms for exendin-4 to affect MSC activities in myocardial infarction, which might present a hint for the same process in bone metabolism. First, exendin-4 activated GLP-1R/cAMP/PKA pathway and attenuated endoplasmic reticulum stress in order to inhibit bone marrow-derived MSC apoptosis mediated by oxygen, glucose, and serum deprivation (57). Second, exendin-4 might regulate MSC growth, mobilization, and survival partly through PI3K/Akt pathway (58). However, further results are needed in order to make an explicit explanation for this issue. Osteoclasts are derived from mature monocytes and macrophages (59); their maturation is regulated by osteoblast-derived cytokines. Among these, osteoprotegerin (OPG), receptor activator for nuclear factor-κB ligand (RANKL), and receptor activator for nuclear factor-κB form a triangular relationship that regulates osteoclast differentiation, activation, and apoptosis. Most of the factors that promote osteoclastogenesis function in osteoclasts perform through enhancing RANKL expression on osteoblasts. Twelve-month-old female Sprague-Dawley aged OVX rats have been observed to increase the mRNA level of OPG while decreasing RANKL mRNA after 16 weeks of exendin-4 use (10). It was also revealed in another study that GLP-1 had more effect on OPG than RANKL in mRNA and protein level of the isolated Wistar rat tibiae (9). Therefore, GLP-1 not only promotes bone formation but also inhibits bone resorption. And to further prove this point, the research by Ma mentioned before focused on aged ovariectomized rats, since aged osteopenia tends to more reduction in bone formation while postmenopausal osteopenia tends to more increase in bone resorption. This study revealed that GLP-1 might have dual anti-osteporosis function on bone tissue (10).

## Conclusion and Expectations

Many scientific investigations have focused on the impact and mechanisms of therapies based on insulin-stimulating hormones, such as GLP-1. The evidence indicates that such therapies may enhance BMD and improve bone quality, but the relationship between GLP-1 and bone fractures is still controversial. Further investigations of the relevant mechanisms have indicated that GLP-1 acts on bone tissue by promoting bone formation and inhibiting bone resorption. Furthermore, the homeostasis of bone formation and resorption is essential to bone health and might be maintained by GLP-1 in normal bones and restored by GLP-1 in unhealthy bones. However, the specific molecular mechanisms responsible for the effects of GLP-1 have still not been fully elucidated. Therefore, although several studies have been conducted, additional multiple-centered RCTs are needed to analyze different parts of bone tissue in patients with different metabolic statuses, being treated with different versions of GLP-1RAs. Elucidating the specific processes and associated molecular pathways will aid in clarifying the impact of GLP-1 on bone metabolism and the mechanisms involved.

## Author Contributions

All the authors contributed equally to the writing, revision, and editing of this manuscript.

## Conflict of Interest Statement

The authors declare that the research was conducted in the absence of any commercial or financial relationships that could be construed as a potential conflict of interest.
